# Supplementary material for: Significance of the inflammatory-immune-nutritional (IINS) score on postoperative survival and recurrence in breast cancer patients: a retrospective study
Source: PeerJ. 2025 Aug 22;13:e19950. doi: 10.7717/peerj.19950 (PMC12377354; doi:10.7717/peerj.19950)
Supplement: Supplemental Information 1 [file peerj-13-19950-s001.doc]

STROBE Statement—checklist of items that should be included in reports of observational studies

|  | Item  No | Recommendation |
| --- | --- | --- |
| **Title and abstract** | 1 | **The significance of inflammation-immunity-nutrition (IINS) score on postoperative survival and recurrence in breast cancer patients** |
|  |  | (1) We analyzed the relationship between the IINS and prognosis of patients with breast cancer.  (2) A high preoperative IINS may be an independent prognostic factor for breast cancer. |
| Introduction | | |
| Background/rationale | 32 | Inflammation, the immune system and nutritional status contribute significantly in tumorigenesis, progression and metastasis. The aim of this study was to evaluate the significance of the Inflammation-Immune-Nutritional Score (IINS) on postoperative survival and recurrence in breast cancer patients and to analyze and compare the Inflammation-Immune-Nutritional Score (IINS), platelet-to-lymphocyte ratio (PLR), and Prognostic Nutritional Index (PNI) in terms of progression-free survival (PFS) and overall survival (OS) in patients with breast cancer (BC) who underwent surgical treatment prognostic value. |
| Objectives | 33 | The aim of this study was to evaluate the significance of the Inflammation-Immune-Nutritional Score (IINS) on postoperative survival and recurrence in breast cancer patients and to analyze and compare the Inflammation-Immune-Nutritional Score (IINS), platelet-to-lymphocyte ratio (PLR), and Prognostic Nutritional Index (PNI) in terms of progression-free survival (PFS) and overall survival (OS) in patients with breast cancer (BC) who underwent surgical treatment prognostic value. |
| Methods | | |
| Study design | 98 | a retrospective study |
| Setting | 98 | Retrospective clinical and pathology data were collected from 200 breast cancer patients undergoing first-time surgical treatment for breast cancer at the Affiliated Hospital of Jiangnan University, between January 2017 and December 2018.The stage of each patient's cancer was determined according to the eighth edition of the American Joint Committee on Cancer (AJCC) staging manual. |
| Participants | 103 | Inclusion criteria were as follows: (1) All patients were pathologically diagnosed as having primary breast cancer; (2) Patients with histologically confirmed nonmetastatic invasive female breast cancer; (3) undergoing radical surgical resection for the first time; (4) with complete clinicopathological and laboratory information. Exclusion criteria: (1) Patients with a history of a malignant tumour or a combination of other primary tumours; (2) Patients with acute and chronic preoperative inflammatory and infectious diseases; (3) Patients treated with radiation before surgery; |
|  |  |  |
| Variables | 43 | IINS was built using the sum of preoperative categorical scores for high-sensitivity C-reactive protein (hs-CRP), lymphocytes (LYM), and albumin (ALB) |
| Data sources/ measurement | 116 | Hospital electronic medical records and clinical data were used to collect pre-surgery blood biochemistry indices and pathology information from breast cancer patients. Blood samples were taken from all of the patients in the week prior to surgery. Body mass index (BMI) was classified in three categories: ≤18.5, 18.5 to 23.9 and >23.9 ^13^. Each patient's cancer stage (including tumour size, axillary lymph node positivity and TNM) was determined using the American Joint Committee on Cancer (AJCC) staging manual, eighth edition ^14^. At baseline before surgery, serum biochemical tests were performed, including laboratory tests (high-sensitivity C-reactive protein, lymphocyte count, thrombocyte count and albumin). Follow-up data were collected using outpatient review and telephone-based follow-up. PFS is the period between the post-operative pathology diagnosis and the physical inspection suggestive of distant metastatic progression. OS is the length of time from the day of the operation to the day of death or the end of the study ^15^. |
| Bias |  | None |
| Study size | 99 | Retrospective clinical and pathology data were collected from 200 breast cancer patients undergoing first-time surgical treatment for breast cancer at the Affiliated Hospital of Jiangnan University, between January 2017 and December 2018. |
| Quantitative variables | 103 | Independent samples t-test or Wilcoxon test was used to compare differences between groups of continuous variables. The chi-square test or Fisher's exact test was used to calculate differences between categorical variables. |
| Statistical methods | 151 | Statistical analysis was undertaken utilizing SPSS 26.0 software. For continuous variables, differences between groups were assessed using the t-test or Wilcoxon signed rank test. Differences between categorical variables are determined using Fisher's exact test or Chi-square test. By predicting PFS and OS for PLR and PNI, the area under the ROC curve (AUC) and the ideal threshold were obtained. Survival curves were depicted utilizing the Kaplan-Meyer technique and differences were compared utilizing the log-rank test ^18^. For univariate and multivariate regression analyses, Cox proportional risk regression models were used. To demonstrate prognostic relationships between patients with different characteristics and the proposed score, subgroup studies were performed. For all two-way adjustments, *P* values < 0.05 were considered statistically significant. |
|  |  |  |

| Results | | |
| --- | --- | --- |
| Participants | 164 | The trial involved 200 patients with breast cancer, all of whom were eventually treated with surgery. Table 1 shows demographic and clinicopathological characteristics. All patients had a median age of 55.5 years (range, 34-75 years). In accordance with AJCC classification, 82 patients (41%) were stage I, 69 (34.5%) were stage II, and 49 (24.5%) were stage III. 7 (3,5 %) had low weight, 94 (47 %) had normal weight and 99 (49,5 %) had excess weight. Regarding tumour size, 101 (50.5%) were T1, 96 (48.0%) were T2, and 3 (1.5%) were T3. 118 (59%) of the axillary lymph nodes were negative, 1-3 (45, 22.5%), 4–9 (22%) and more than 10 (7.5%) were positive. The ER status was negative in 130 patients (65%) who had a positive ER status and in 70 patients (35%) who had a negative ER status. A total of 98 patients (49%) had a positive PR status and 102 (51%) had a negative status. For the Her-2 status, 163 (81.5%) had a positive result and 37 (18.5%) had a negative result. Overall, 77 patients (38.5%) relapsed, and 43 patients (21.5%) died. The average follow-up was 46 months. |
| Descriptive data | 46 | (a) Age, BMI, Tumor size, TNM stage, Node positivity, ER, PR, HER-2, PLR,PNI,IINS |
| Outcome data |  | This is a Retrospective Study, this item is not applicable. |
| Main results | 49 | The median age of the patients was 55.5 years (range 34-75 years). In progression-free survival, the areas under the IINS, PLR, and PNI curves were as follows: IINS: 0.735, HR (95% CI) 0.037 (0.662-0.809), PLR: 0.724, HR (95% CI) 0.036 (0.655-0.794), PNI: 0.694, HR (95% CI) 0.038 (0.619-0.769). In overall survival, the areas under the curves of IINS, PLR, and PNI were as follows: IINS: 0.738, HR (95% CI) 0.049 (0.642-0.834), PLR: 0.700, HR (95% CI) 0.039 (0.623-0.777), and PNI: 0.713 with HR (95% CI) 0.050 (0.615-0.811). According to the findings, among patients with resectable breast cancer, preoperative IINS may be the most accurate indicator of both overall survival and progression-free survival. |
|  |  | Tumor size (＜2cm、＞2cm,＜5cm、＞5cm). Node positivity (0、1~3、4~9、＞10) ER、PR、HER-2（Positive、Negative） |
| Other analyses | 157 | For univariate and multivariate regression analyses, Cox proportional risk regression models were used. To demonstrate prognostic relationships between patients with different characteristics and the proposed score, subgroup studies were performed. |
| Discussion | | |
| Key results | 58 | IINS may be a dependable marker for predicting postoperative survival in patients with breast cancer, and its prognostic value may be higher than that of traditional markers. |
| Limitations | 255 | The key advantage of the current study is that we employed a newly developed indicator, IINS, which was based on hs-CRP, LYM, and ALB and may perform better than some of the conventional indicators generated from inflammatory, immunological, and nutritional components ^28^ . However, there are some limitations to our study: First off all, despite the fact that the IINS threshold value was determined from clinical reference values, there were variations in sample size and patient selection criteria across studies, which will inevitably bias the results. Although we built a validation cohort for internal validation, the lack of external validation compromises the robustness and generalizability of the study. Secondly, this study involved a relatively small sample size and included patients from only one center, and the conclusions may be potentially biased; To confirm our findings, multicentre, large-scale prospective studies are needed. Thirdly, the relationship between postoperative IINS changes and the prognosis of these patients has not yet been investigated in our study, which focused on the prognostic significance of preoperative IINS. Longitudinal studies should be performed in the future to verify the prognostic significance of the fact that many patients received multiple treatments for tumour recurrence during the follow-up period, which also influenced prognosis. Finally, our study included a small amount of clinical data that could be used in the future to characterize the dynamics of patients as their disease progresses by statistically analyzing their comorbidities and other biochemical data, and the results may be more instructive in the future. |
| Interpretation |  |  |
| Generalisability | 278 | According to our findings, preoperative IINS may be a potent prognostic predictor in patients with operable breast cancer. In the case of prognosis in particular, IINS is superior to PLR or PNI. Suggesting that it may give a straightforward way of identifying individuals with bad prognosis and the chance to lead therapy and follow-up efforts to improve their status. |
| Other information | | |
| Funding | 295 | This work was supported by the Wuxi Taihu Lake Talent Plan, Support for Leading Talents in Medical and Health Professions (Mading academician, 4532001THMD), Wu Jinping Medical Foundation（320.6750.2024-6-100), General Project of Wuxi Health Commission（M202408, M202418） |

*Give information separately for cases and controls in case-control studies and, if applicable, for exposed and unexposed groups in cohort and cross-sectional studies.

**Note:** An Explanation and Elaboration article discusses each checklist item and gives methodological background and published examples of transparent reporting. The STROBE checklist is best used in conjunction with this article (freely available on the Web sites of PLoS Medicine at http://www.plosmedicine.org/, Annals of Internal Medicine at http://www.annals.org/, and Epidemiology at http://www.epidem.com/). Information on the STROBE Initiative is available at www.strobe-statement.org.
