# Supplementary material for: Significance of the inflammatory-immune-nutritional (IINS) score on postoperative survival and recurrence in breast cancer patients: a retrospective study
Source: PeerJ. 2025 Aug 22;13:e19950. doi: 10.7717/peerj.19950 (PMC12377354; doi:10.7717/peerj.19950)
Supplement: Supplemental Information 2 — Abbreviations AUC, Area under the ROC Curve; IINS, inflammation-immunity-nutrition score; PLR, platelet count to lymphocyte count ratio; PNI, nutrient index. [file peerj-13-19950-s002.docx]

**Supplementary Table 1** ROC analysis of breast cancer patients

|  |  | **AUC (95%CI)** | | |  |
| --- | --- | --- | --- | --- | --- |
| **Indices** | **PFS** | | **P** | **OS** | **P** |
| PLR | 0.724 (0.655-0.794) | | 0.025 | 0.700 (0.623-0.777) | 0.034 |
| PNI | 0.694 (0.619-0.769) | | 0.046 | 0.713 (0.615-0.811) | 0.012 |
| IINS | 0.735 (0.622-0.809) | | ＜0.001 | 0.738 (0.642-0.834) | ＜0.001 |

***Abbreviations*** *AUC, Area under the ROC Curve; IINS, inflammation-immunity-nutrition score; PLR, platelet count to lymphocyte count ratio; PNI, nutrient index.*
