# Supplementary material for: Significance of the inflammatory-immune-nutritional (IINS) score on postoperative survival and recurrence in breast cancer patients: a retrospective study
Source: PeerJ. 2025 Aug 22;13:e19950. doi: 10.7717/peerj.19950 (PMC12377354; doi:10.7717/peerj.19950)
Supplement: Supplemental Information 3 — PPV, positive predictive value; NPV, negative predictive value; PFS, progression-free survival; OS, Overall Survival. [file peerj-13-19950-s003.docx]

Supplementary Table 2: Comparison between ROC curves of IINS, PNI and PLR.

| **Indices** | | **PFS** | | | | **OS** | | | |
| --- | --- | --- | --- | --- | --- | --- | --- | --- | --- |
|  | sensitivity | specificity | PPV (%) | NPV (%) | sensitivity | specificity | PPV (%) | NPV (%) |  |
| PLR | 0.831 | 0.610 | 0.571 | 0.693 | 0.860 | 0.535 | 0.336 | 0.178 |  |
| PNI | 0.756 | 0.545 | 0.509 | 0.583 | 0.847 | 0.581 | 0.356 | 0.237 |  |
| IINS | 0.584 | 0.805 | 0.652 | 0.545 | 0.698 | 0.726 | 0.411 | 0.397 |  |

**Notes:**

*PPV, positive predictive value; NPV, negative predictive value; PFS,* ***progression-free survival****; OS,* *Overall Survival.*
