# Supplementary material for: Significance of the inflammatory-immune-nutritional (IINS) score on postoperative survival and recurrence in breast cancer patients: a retrospective study
Source: PeerJ. 2025 Aug 22;13:e19950. doi: 10.7717/peerj.19950 (PMC12377354; doi:10.7717/peerj.19950)
Supplement: Supplemental Information 4 [file peerj-13-19950-s004.docx]

ER：

1： positive 2: negative

PR:

1： positive 2: negative

HER-2

1： positive 2: negative

PFS:

0: without 1: with

OS:

0: without 1: with

TNM:

1: I stage 2: II stage 3: III stage 4: IV stage

Treatment:

1: chemotherapy 2: radiotherapy 3: Other treatments
